# The electronic structure and deexcitation pathways of an isolated metalloporphyrin ion resolved by metal L-edge spectroscopy†

**DOI:** 10.1039/d0sc06591a

**Published:** 2021-02-03

**Authors:** Kaja Schubert, Meiyuan Guo, Kaan Atak, Simon Dörner, Christine Bülow, Bernd von Issendorff, Stephan Klumpp, J. Tobias Lau, Piter S. Miedema, Thomas Schlathölter, Simone Techert, Martin Timm, Xin Wang, Vicente Zamudio-Bayer, Lucas Schwob, Sadia Bari

**Affiliations:** Deutsches Elektronen-Synchrotron DESY 22607 Hamburg Germany kaja.schubert@desy.de sadia.bari@desy.de; Division of Chemical Physics, Chemical Center, Lund University SE-221 00 Lund Sweden; Abteilung für Hochempfindliche Röntgenspektroskopie, Helmholtz-Zentrum Berlin für Materialien und Energie 12489 Berlin Germany; Physikalisches Institut, Albert-Ludwigs-Universität Freiburg 79104 Freiburg Germany; Zernike Institute for Advanced Materials, University of Groningen 9747 AG Groningen The Netherlands; Institut für Röntgenphysik, Georg-August-Universität Göttingen 37077 Göttingen Germany

## Abstract

The local electronic structure of the metal-active site and the deexcitation pathways of metalloporphyrins are crucial for numerous applications but difficult to access by commonly employed techniques. Here, we applied near-edge X-ray absorption mass spectrometry and quantum-mechanical restricted active space calculations to investigate the electronic structure of the metal-active site of the isolated cobalt(iii) protoporphyrin IX cation (CoPPIX^+^) and its deexcitation pathways upon resonant absorption at the cobalt L-edge. The experiments were carried out in the gas phase, thus allowing for control over the chemical state and molecular environment of the metalloporphyrin. The obtained mass spectra reveal that resonant excitations of CoPPIX^+^ at the cobalt L_3_-edge lead predominantly to the formation of the intact radical dication and doubly charged fragments through losses of charged and neutral side chains from the macrocycle. The comparison between experiment and theory shows that CoPPIX^+^ is in a ^3^A_2g_ triplet ground state and that competing excitations to metal-centred non-bonding and antibonding σ* molecular orbitals lead to distinct deexcitation pathways.

## Abstract 2

Near-edge X-ray absorption mass spectrometry (NEXAMS) and restricted active space (RAS) quantum mechanical calculations at the metal L-edge reveal the electronic structure and orbital-specific deexcitation pathways of gas-phase metalloporphyrins.

## Graphical abstract

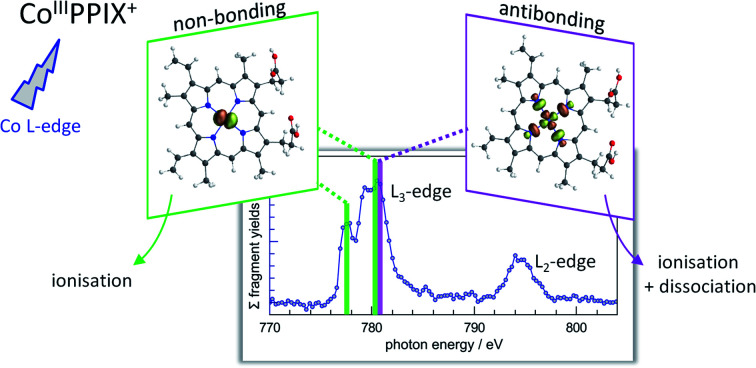

## Introduction

Metalloporphyrins are very common organometallic molecules that play key roles in nature: heme (iron protoporphyrin IX) is the prosthetic group in hemoglobin and is responsible for oxygen transport,^1^ chlorophyll A (a magnesium-containing porphyrin) is involved in the photosynthesis of plants^2^ and cobalt protoporphyrin IX is an inducer of hemeoxygenase-1, an enzyme that catalyses the heme catabolism.^3^ Their ability to bind axial ligands such as oxygen, to act as electron transfer agents as well as their light absorption properties make metalloporphyrins promising candidates for many applications including photosensitisers,^4,5^ radiolabelled molecular imaging,^6^ solar cells,^7,8^ colorimetric sensors,^9,10^ and catalysts.^11^

**Fig. 1:**
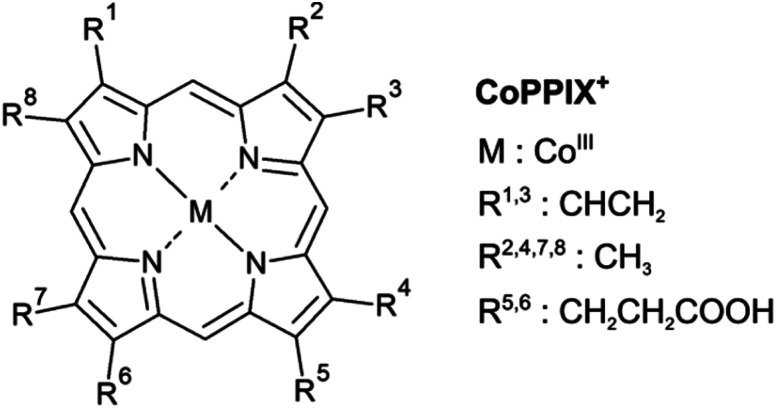
Structure of metalloporphyrins. The peripheral side chains of the cobalt(iii) protoporphyrin IX cation (CoPPIX^+^) are listed on the right.

Metalloporphyrins consist of a metal ion coordinated by four nitrogen atoms of a porphyrin macrocycle (Fig. 1). They show a strong absorption in the UV/vis, caused by resonant π–π* transitions in the porphyrin macrocycle, namely the Soret and Q bands.^12^ The electronic structure of metalloporphyrins is crucial for their functionality and is determined by *e.g.* the axial ligands and the spin and oxidation state of the metal centre. In hemoproteins, for instance, oxygen transport involves the switching between high-spin and low-spin states and electron transfer is accomplished by reversible oxidation and reduction of the metal centre.^13^ Getting deeper knowledge of the metalloporphyrin electronic structure is therefore essential for the understanding of their functionality and for the synthesis of new porphyrins with tailored properties for new applications. In some applications, metalloporphyrin-based materials and metalloporphyrin-containing proteins may be subject to ionising radiation. Fundamental insight into dissociation pathways/mechanisms of metalloporphyrins can, here, improve the comprehension of the radiation damage at the macroscopic scale.

The high reactivity of metalloporphyrins towards molecules such as oxygen is crucial in nature but complicates studying non-oxidised metalloporphyrins in the condensed phase.^14^ In addition, many porphyrins tend to form aggregates in solution.^15,16^ In contrast, gas-phase experiments on isolated ionic metalloporphyrins, as produced by electrospray ionisation sources, provide control over stoichiometry,^17^ axial ligands^18–20^ and the oxidation state of metalloporphyrins,^21–23^ thus allowing the study of fundamental properties of the molecules in well-defined states.

Previous mass-spectrometric studies on electrosprayed metal protoporphyrin IX ions employed collision-induced dissociation (CID),^24–27^ electron-capture-induced dissociation (ECD)^28^ and laser-induced dissociation (LID)^18,25,26,29–32^ following absorption at the Soret and Q bands to investigate their dissociation processes. Moreover, it has been shown for gas-phase heme^+^ that photoexcitation in the UV/vis region can be followed by dissociation *via* two kinetically competing channels on a microsecond to millisecond timescale.^25,30^ The ions can undergo (1) direct internal conversion from the excited state to the vibrationally excited ground state, followed by intramolecular ro-vibrational redistribution (IVR) of the internal energy and statistical dissociation or (2) intersystem crossing to a lower-lying state with a higher spin followed by another spin flip to the vibrationally excited ground state and subsequent IVR and dissociation. The main fragmentation channel in CID and LID experiments of protoporphyrin IX (PPIX) molecules with and without metal is the β-cleavage, resulting in single and double losses of the acetic side chains CH_2_COOH˙.^24–27^ Additional peripheral side-chain losses are minor fragmentation channels.

In the UV/vis energy region, studying the active site of metalloporphyrins is hampered by the strong π–π* transitions in the macrocycle which obscure the dipole-forbidden and thus weak d–d electronic transitions.^33^ L_2,3_-edge spectroscopy (2p → 3d transitions, further referred to as L-edge spectroscopy) of 3d transition metal elements using soft X-rays, in contrast, directly allows probing molecular orbitals with 3d character^34^ and is therefore a powerful tool to study the electronic structure of the metal-active site in metalloporphyrins.^14,35–42^ The combination of 3^rd^ generation synchrotron sources – able to deliver the required photon energies with a sufficient photon flux – with electrospray ionisation sources and mass spectrometry, also referred to as near-edge X-ray absorption mass spectrometry (NEXAMS), has been mainly employed to investigate peptides and proteins in the gas phase.^43–45^

NEXAMS, as an ion yield absorption spectroscopy technique, provides the integrated ion yield of all product ions for each photon energy step, referred to as total ion yield (TIY). The TIY is proportional to the X-ray absorption of the system if deexcitation by fluorescence can be neglected.^46^ This is indeed the case for the 2p core-hole decay of 3d transition metals for which Auger processes dominate and contributions from fluorescence are <1%.^47^ In addition, NEXAMS provides partial ion yields (PIYs) for individual photo products, which can reveal correlations between deexcitation pathways and electronic transitions. PIY spectra of ferrocene after iron 2p inner-shell excitation, for instance, revealed that excitations to molecular orbitals with predominantly iron-3d character in ferrocene lead to more efficient ionisation, whereas excitations to ligand-centred π* orbitals lead to more efficient fragmentation.^48^ Iron pentacarbonyl, in contrast, does not exhibit such an orbital- or state-specific behaviour, which could be attributed to a different degree of core-hole screening for the different ligands or to a different degree of charge rearrangements in the Auger final states.^48^

In the 3d transition metal L-edge energy regime the X-ray spectral features are usually shaped by a multitude of spectral effects, such as spin-orbit coupling, multiplet effects and selection rules, which make the spectrum challenging to interpret. The individual transitions are further broadened by the lifetime of the core-hole state which can lead to complicated peak shapes that usually cannot easily be decomposed into spectral contributions. Therefore, calculations of metal L-edge X-ray absorption spectrum (XAS) are required for interpretation of metal L-edge spectra. Moreover, the validation of such theoretical models relies on the comparison with benchmark experiments which investigations on gas-phase isolated systems can provide.

The aim of this study is to assess the capability of NEXAMS for exploring the electronic structure of the metal-active site of an isolated “model” metalloporphyrin, here the cobalt(iii) protoporphyrin IX cation, CoPPIX^+^. In the present paper, NEXAMS at the cobalt L-edge was combined with quantum-chemical restricted active space (RAS) calculations, which have shown their validity on 3d transition metal complexes,^49–56^ to unravel the electronic structure of isolated CoPPIX^+^. In the following, we discuss the fragmentation pathways of CoPPIX^+^ subsequent to single and multiple ionisation after resonant photoexcitation at the cobalt L_3_-edge. TIY and PIYs are later interpreted with the help of quantum-chemical RAS calculations.

## Methods

### Experiment

Protoporphyrin IX cobalt chloride, methanol, dichloromethane and formic acid were purchased from Merck and were used without further purification.

In the present study a home-built electrospray ionisation source was interfaced with the Nanocluster trap end station^57,58^ of the UE52_PGM beamline at the BESSY II synchrotron (HZB Berlin, Germany). Cobalt(iii) protoporphyrin IX cations were formed by electrospray ionisation of a 30 μM protoporphyrin IX cobalt chloride solution in methanol and dichloromethane (1 : 1 in volume) with 1 vol% formic acid. Precursor ions were selected by their mass-to-charge ratio with a quadrupole mass filter and were deflected to the photon beamline axis with an electrostatic bender. The ions were accumulated in a cryogenic linear ion trap (*T* = 18 K) with helium buffer gas before being exposed to the soft X-ray photons. The exit slits and grating of the monochromator were set to an energy bandwidth of 680 meV at 770 eV and energy scans across the cobalt L-absorption edge (770–804 eV) were performed with 300 meV steps. The photon flux was measured with a calibrated GaAsP photodiode behind the interaction zone. Cationic photo products were analysed by their mass-to-charge ratio using a reflectron time-of-flight mass spectrometer and were detected with a micro-channel plate detector. The mass calibration was based on the well-known photofragmentation mass spectrum of leucine enkephalin (see ESI†). The mass resolution varied due to the specificity of the ion trap and spectrometer and was as follows: *m*/Δ*m* = 1600 at *m*/*z* 309.6, *m*/Δ*m* = 1350 at *m*/*z* 268.6 and *m*/Δ*m* = 900 at *m*/*z* 249.5. The mass calibration and resolution allowed us to distinguish the isotopic pattern of the photo product CoPPIX^2+^. Comparison with the theoretical isotopic pattern (Fig. ESI-1†) confirmed that the precursor ions are in the cationic Co^III^PPIX^+^ (*m*/*z* 619.6) and not the protonated [Co^II^PPIX + H]^+^ (*m*/*z* 620.6) form.

All obtained mass spectra were normalised to the photon flux. To distinguish the effect of resonant absorption at the cobalt 2p core level from non-resonant absorption in the porphyrin ring and side chains of CoPPIX^+^, a mass spectrum below the L_3_-edge was subtracted from the mass spectra presented here. The below-edge mass spectrum was determined as the average of the eight mass spectra obtained between 770 and 772 eV.

**Fig. 2:**
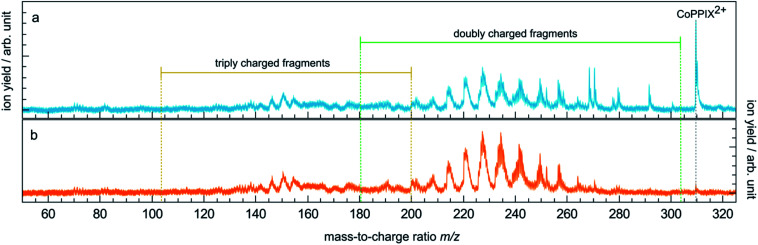
(a) Mass spectrum of CoPPIX^+^ upon resonant absorption at 780.5 eV at the cobalt L_3_-edge after subtraction of the below-edge mass spectrum (see Experimental section). (b) Below-edge mass spectrum of CoPPIX^+^. The mass ranges of doubly and triply charged fragments are indicated in green and yellow, respectively.

The TIY was given by integrating the mass spectra over the range 145 < *m*/*z* < 315 (see Fig. 2) for each photon energy step. The PIYs were determined by integrating the ion yield of each individual photo product per energy step. Contributions from isotopic peaks were not considered.

### Computation

The molecular geometry was optimised using density functional theory (DFT) with the B3LYP functional and the 6-311g* basis set using Gaussian 09,^59^ the structure and coordinates are available in the ESI.† The energies of different low-lying states were calculated using the complete active space self-consistent field (CASSCF) method. The active space, shown in Fig. ESI-6,† is composed of five cobalt 3d-character orbitals together with one porphyrin-character σ donation orbital and five empty cobalt 4d-character orbitals. The energy calculations of low-lying states enable us to assign the ground state as a ^3^A_2g_ triplet state with the electron configuration d_*xy*_^2^d_*z*^2^_^2^d_*xz*_^1^d_*yz*_^1^d_*x*^2^−*y*^2^_^0^. The energies together with the schematic representations of selected low-lying states are available in Fig. ESI-7.† Under the spin orbit-coupling scheme the ground state has a ^3^A_2g_ character with a mixture of other low-lying states. The quantitative description of the state mixing can depend on the calculation strategy, such as the basis set and the active space, and is challenging without evident experimental determination. However, the confirmation of the ground-state electronic structure can be further complemented through the simulation of cobalt metal L-edge XAS and comparison to experimental data. For the cobalt L-edge XAS calculation, the restricted active space SCF (RASSCF) approach is used. In addition to the active orbitals for the CASSCF calculation, the cobalt 2p core orbitals are placed in the RAS1 subspace. The highly excited states (HEXS) technique implemented in the OpenMolcas^60^ is used for the core-excited states calculation.^61–63^ Up to 60 core-excited states per spin were calculated, which gave a total number of 540 spin-orbit core-excited states. The spectrum dependences on the number of core-excited states are shown in Fig. ESI-8.† Scalar relativistic effects have been included by using a second-order Douglas–Kroll–Hess Hamiltonian^64,65^ in combination with the ANO-RCC-VDZ basis set and the use of a Cholesky decomposition approach to approximate the two-electron integrals.^66^ For comparison to the experimental spectrum, the simulated RAS spectrum is plotted using a Lorentzian broadening with a full-width-at-half-maximum (FWHM) of 0.15 and 0.65 eV for the L_3_- and L_2_-edge respectively, further convoluted with a Gaussian broadening of 0.3 eV. An energy shift of −6.22 eV is applied to the calculated cobalt L-edge XAS to match the experimental spectrum. The similarity between the calculated XAS spectra and the experimental spectrum were analysed through a weighted cross-correlation function.^67^ The similarity scores can be found in Table ESI-3.†

## Results and discussion

### Fragmentation of CoPPIX^+^ at the cobalt L_3_-edge

The mass spectrum obtained for CoPPIX^+^ in the mass range 50 < *m*/*z* < 325 after resonant photoabsorption at 780.5 eV at the cobalt L_3_-edge is presented in Fig. 2a. In the energy region at the cobalt L-edge studied here (770–804 eV), we observe the strongest TIY at 780.5 eV (see text below) and we therefore discuss the photofragmentation based on the mass spectrum at this energy. The mass spectrum is dominated by the intact radical dication CoPPIX^2+^ at *m*/*z* 309.6 and by a number of doubly charged fragments at *m*/*z* < 309.6. The high stability of the doubly charged metalloporphyrin is remarkable, similar to as has been observed in the case of ruthenium octaethyl porphyrin ligated to CO upon sequential optical multiphoton ionisation.^68^ We assume that peaks at *m*/*z* < 206.4, with *m*/*z* 206.4 corresponding to the intact triply charged CoPPIX^3+^, are mainly due to triply charged fragments. The observed photo products will be discussed in more detail later in this section.

**Scheme 1:**
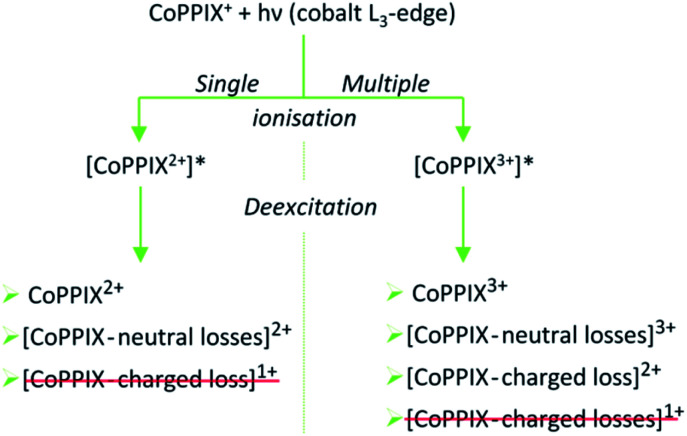
Possible photo products upon single and multiple ionisation after resonant cobalt 2p inner-shell excitation of CoPPIX^+^ at 780.5 eV at the cobalt L_3_-edge. Photo products which are not observed in the mass spectrum in Fig. 2 and in ESI-2† are crossed out in red.

After creation of a cobalt 2p inner-shell hole in CoPPIX^+^, deexcitation proceeds by Auger and radiative processes. The single Auger decay creates one additional positive charge in the molecule, whereas other autoionisation processes such as sequential or multiple Auger processes or shake-up processes lead to higher ionisation of the molecule. Single and multiple ionisation can be followed by different deexcitation pathways and will be discussed separately in the following. An overview of these possible processes is shown in Scheme 1.

### Single ionisation

**Fig. 3:**
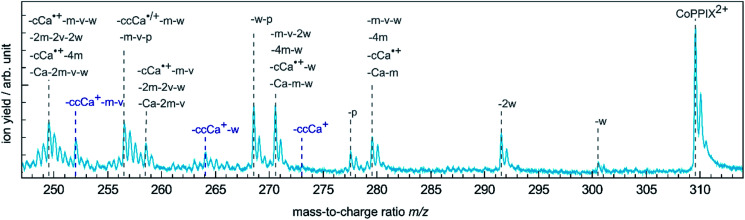
Zoom into the mass spectrum of CoPPIX^+^ upon resonant absorption at 780.5 eV at the cobalt L_3_-edge after subtraction of the below-edge mass spectrum (see Experimental section, see Fig. 2a). The assignments of the observed peaks are indicated.

The single Auger process, where an electron from a molecular orbital with 3d character fills the cobalt 2p core hole and an electron from a molecular orbital with 3d character leaves the molecule, leads to the formation of the excited radical dication [CoPPIX^2+^]*. The molecule may stay intact or fragment by neutral or charged fragment losses (or a combination of both). Since no singly charged photo fragments are observed at *m*/*z* > 320 (Fig. ESI-2†), charged fragment losses from excited [CoPPIX^2+^]* can be excluded. Single Auger decay in CoPPIX^+^ leads to the intact radical dication CoPPIX^2+^ and doubly charged fragments formed by neutral losses from excited [CoPPIX^2+^]*. To discuss the observed doubly charged fragments in more detail we show in Fig. 3 a zoom of the mass spectrum of Fig. 2a in the mass range 247 < *m*/*z* < 314.

**Fig. 4:**
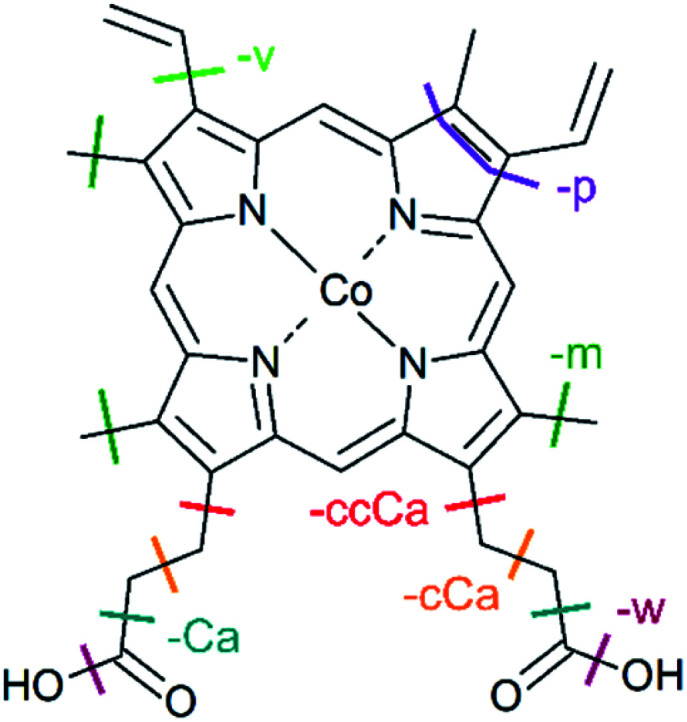
Structure of CoPPIX^+^. The indicated bond cleavages lead to the observed fragments in Fig. 2 and in Fig. 3.

The doubly charged fragments arise essentially from single and multiple side-chain losses from the ionised metalloporphyrin, as indicated in Fig. 4. In Fig. 3, the peak corresponding to the intact radical dication is observed at *m*/*z* 309.6. Peaks at 300.6 and 291.6 are assigned to the losses of one and two neutral H_2_O groups from CoPPIX^2+^, respectively. H_2_O loss from metalloporphyrins was observed in both, CID^24^ and LID^25^ experiments as a minor fragmentation channel. Peaks at lower *m*/*z* in Fig. 3 are assigned to multiple neutral side-chain losses of methyl (CH_3_˙ = m, −15 u), water (H_2_O = w, −18 u), vinyl (CHCH_2_˙ = v, −27 u) and carboxylic groups (CH_2_CH_2_COOH˙ = ccCa˙, −73 u; CH_2_COOH˙ = cCa˙, −59 u; COOH˙ = Ca, −45 u). Similar side-chain losses were observed as neutral losses in CID^24–27^ and in LID^18,25,26,29,30^ experiments, although we observe overall a higher number of fragments from cascade dissociations after core-hole relaxation. Interestingly, single and double losses of CH_2_COOH˙ groups from CoPPIX^2+^ (*m*/*z* 280.1 and 250.5, respectively), which are the two main fragmentation channels of metal protoporphyrin IX cations in LID and CID experiments,^27^ are only weak fragmentation channels upon cobalt 2p inner-shell excitation of CoPPIX^+^.

In addition, we observe peaks at *m*/*z* 277.6 and 268.6, corresponding to losses of 64 u and 82 u, which do not match any combinations of the commonly observed side-chain losses. We assign the *m*/*z* 277.6 peak to the loss of a pentadiyne group (C_5_H_4_ = p) and the *m*/*z* 268.6 peak to the additional loss of H_2_O (-w-p in Fig. 3). C_5_H_4_ fragments could be formed by one vinyl group, one methyl group and two carbon atoms of the porphyrin ring, accompanied with the transfer of two hydrogen atoms from the side chains to the two carbon atoms adjacent to a nitrogen atom of one pyrrole subunit (see Fig. 4).

Table ESI-1† lists possible assignments for the multiple side-chain losses. However, losses of a cobalt atom or ion were not considered since demetallation can be excluded from comparison with mass spectra that we obtained for other metalloporphyrins after resonant photoexcitation at the metal L_3_-edge (data not shown here).

To some extend the observed losses are similar to those produced by collisional activation, which suggest that similar dissociation mechanisms as after collisional activation are involved after the cobalt 2p core-hole relaxation in CoPPIX^+^. This would involve internal conversion of the electronically to the vibrationally excited molecule, followed by IVR of the internal energy, ultimately leading to dissociation. The overall stronger cascade dissociations we observe can be the result of a higher internal energy in the system deposited after cobalt 2p inner-shell excitation compared to CID and to UV/vis absorption at the Q and Soret bands. In addition, the observation of fragments different from the common CID/LID losses suggests that other dissociation processes are involved in the present study. One possible explanation is that upon ionisation, the additional radical on the porphyrin may open up new fragmentation pathways with low energy barriers. For peptides it is for instance known that such radical cationic species undergo facile cascade dissociations.^69^

### Multiple ionisation

While the single Auger decay creates one additional charge in the molecule, other autoionisation processes result in at least two additional charges leading to the formation of excited highly charged [CoPPIX^*z*≥3+^]* (see Scheme 1). For resonant iron 2p inner-shell excitation of Fe^2+^ ions with 3d^6^ electrons, similar to cobalt ions in CoPPIX^+^, double ionisation is the main deexcitation process.^70^ Therefore, we discuss the case *z* = 3, corresponding to double ionisation of CoPPIX^+^, here. Deexcitation of [CoPPIX^3+^]* may lead to the formation of CoPPIX^3+^ and singly to triply charged CoPPIX fragments by single or multiple losses of neutral and/or charged fragments. Indeed, we observe several peaks as minor contributions in the mass spectrum, which are unambiguously assigned to triply charged fragments (Fig. 2a at 145 < *m*/*z* < 160). These fragments could be formed from neutral losses of excited [CoPPIX^3+^]*. Formation of the intact CoPPIX^3+^ at *m*/*z* 206.4 cannot be resolved in the present data but cannot be strictly excluded neither. However, the contribution of CoPPIX^3+^ in the mass spectrum would be very low compared to the doubly charged fragments (Fig. 2a). Moreover, since no singly charged fragments at *m*/*z* > 320 are formed (Fig. ESI-2†) double charged fragment losses from excited [CoPPIX^3+^]* can be ruled out. Although charged fragment losses from excited [CoPPIX^2+^]* are not observed, the stronger Coulombic repulsion in excited highly charged [CoPPIX^*z*≥3+^]* may cause single charged fragment losses in the latter case.

Indeed, mass spectra acquired in the energy region of the carbon K-edge of CoPPIX^+^ (Fig. ESI-3†) reveal that the doubly charged fragments at *m*/*z* 273.1, *m*/*z* 264.1 and *m*/*z* 252.0 are almost exclusively formed upon photoionisation above the carbon 1s ionisation threshold compared to resonant excitation. Above the carbon 1s ionisation edge of CoPPIX^+^, photoionisation and subsequent autoionisation processes lead to the formation of excited [CoPPIX^3+^]* followed by further deexcitation. Therefore, fragments at *m*/*z* 273.1, *m*/*z* 264.1 and *m*/*z* 252.0 can be unambiguously assigned to charged fragment losses from excited [CoPPIX^3+^]*. In particular charged carboxylic groups, namely CH_2_CH_2_COOH^+^ (73 u) and CH_3_COOH˙^+^ (60 u), are known as the main products from electron impact ionisation of long-chain carboxylic acids^71^ and could contribute to charged fragment losses from excited [CoPPIX^*z*≥3+^]*. With this assumption we assign the fragment series at *m*/*z* 273.1, 264.1 and 252.0 to the loss of ccCa^+^, ccCa^+^ + w and ccCa^+^ + m + v, respectively (see Fig. 3). Noteworthy, most of the fragments at *m*/*z* < 267 can be attributed to losses containing one or two cCa or ccCa groups. For ccCa˙, the corresponding charged fragment with ccCa^+^ would fall at the same *m*/*z* and could contribute to these peaks.

To further elucidate if the observed doubly charged fragments in Fig. 2a arise from charged or neutral fragment losses, we show in Fig. 2b a mass spectrum acquired below the L_3_-edge (see Experimental section). Below the L_3_-edge, non-resonant photoabsorption in the valence shell and in the carbon, nitrogen and oxygen K-shells dominates in CoPPIX^+^. Here, photoabsorption of CoPPIX^+^ leads predominantly to photoionisation and subsequent autoionisation, resulting in at least two additional charges in the molecule. Accordingly, the yield of the CoPPIX^2+^ dication at *m*/*z* 309.6 in Fig. 2b is very weak and arises from photoionisation in high-lying valence states. We observe various doubly charged fragments in Fig. 2b at *m*/*z* < 267, which can only arise from charged side-chain losses of highly excited [CoPPIX^*z*≥3+^]*. The peak pattern in the mass spectra at *m*/*z* < 267 below (Fig. 2b) and on-below (Fig. 2a) the L_3_-edge is similar, indicating that these fragments arise from similar processes, namely autoionisation leading to multiple ionisation with subsequent charged fragment loss.

To summarise, doubly charged fragments formed by a single Auger decay with subsequent neutral losses and doubly charged fragments formed by other autoionisation processes with subsequent charged (and neutral) fragment losses can contribute to the same peaks at *m*/*z* < 267 in one mass spectrum. Most of the fragments at *m*/*z* > 267, in contrast, are formed upon resonant absorption (Fig. 2a), but not in the energy region below the L_3_-edge (Fig. 2b). We therefore conclude that these fragments are mainly produced from neutral losses of the CoPPIX^2+^ dication in an excited state, which is only formed upon resonant absorption.

### Total ion yield (TIY) spectrum of CoPPIX^+^

**Fig. 5:**
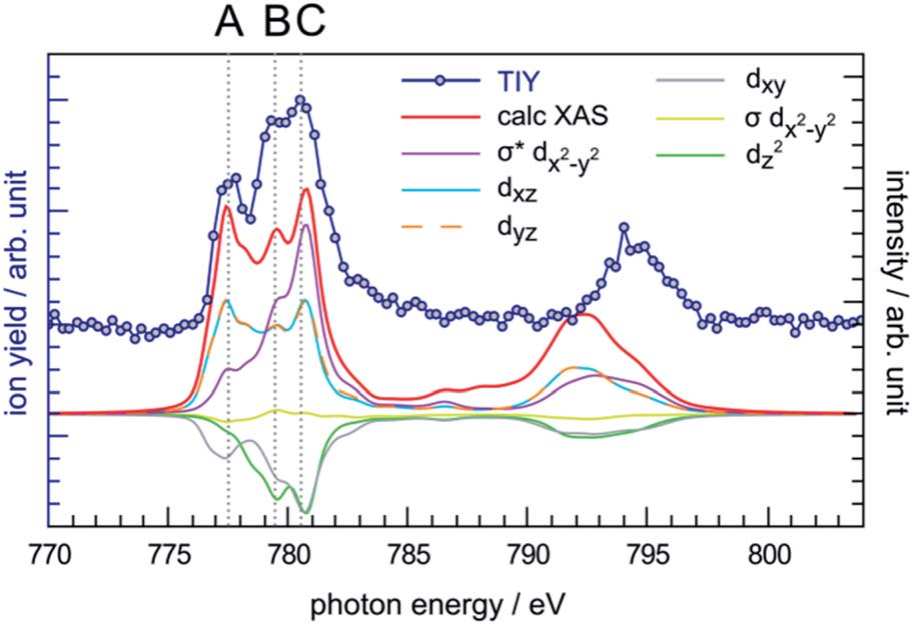
Comparison between the experimental TIY spectrum and the calculated XAS spectrum of CoPPIX^+^ at the cobalt L-edge, including the orbital-contribution analysis. The positive and negative values of the orbital contribution curves represent electron gain and loss in the transitions from initial state to core-excited states, the constant one-electron loss in the 2p core orbital is not shown.^43^

The photon energy range 770–804 eV covers the resonances associated with the excitation of cobalt 2p electrons to molecular orbitals with 3d character. The cobalt L-edge XAS splits into the L_3_- and L_2_-edges by the core 2p spin-orbit coupling. In the following discussion we focus on the L_3_-edge as it is better resolved compared to the L_2_-edge due to the core-hole lifetime difference.^72^ The experimental L_3_-edge has a broad feature and consists of three peaks located at 777.4 eV, 779.4 eV and 780.5 eV, labelled as peak A, B and C, respectively (Fig. 5).

The cobalt L-edge XAS of CoPPIX^+^ were calculated based on the initial states (^3^A_2g_, ^3^E_g_, ^3^B_1g_, and ^5^A_1g_) with different electron configurations, suggesting that the multiplet shape of the spectrum refers to an intermediate-spin state as for iron in iron phthalocyanine, where a mixture of intermediate states contributes to the ground state.^73^ Schematic representations of the selected electron configurations together with their corresponding XAS are available in the ESI.† The ground state has a triplet ^3^A_2g_ character with a d_*xy*_^2^d_*z*^2^_^2^d_*xz*_^1^d_*yz*_^1^d_*x*^2^−*y*^2^_^0^ configuration with a mixture of other low-lying states (see Computational section). As shown in Fig. 5 and in the similarity score Table ESI-3,† the calculated XAS with this configuration reproduces the experimental spectral features with the best agreement, confirming our assignment of the ground state. The relative intensities of peaks B and C are well reproduced, while the intensity of peak A is overestimated relative to the other peaks. Nevertheless, this is not surprising as PIYs for photo products exhibit differences in the intensity of peak A (see text below) and as the sensitivity of the spectrometer is different towards photo products with different *m*/*z*'s (see Experimental section).

The calculated cobalt L-edge XAS is further analysed in terms of the core-excited spin state contributions (Fig. ESI-10†) and molecular orbital contributions (Fig. 5). The decomposition of orbital contributions to the calculated spectrum in Fig. 5 provides a clearer picture of intensity origins of the observed peaks. In the orbital contribution analysis, only the spin-free states contributing to the final intensity of a transition between spin-orbit coupling states were considered. Each orbital contribution curve is the sum of all transitions we calculated for the spectrum (see Fig. ESI-6† for representation of the molecular orbitals). Peak A gains most of its intensity from excitations to the non-bonding d_*xz*_/d_*yz*_ orbitals strongly localised on the cobalt ion with some intensity from excitations to the antibonding σ* d_*x*^2^−*y*^2^_ orbital where the electron density is partly delocalised towards the nitrogen atoms. Peak C has most of its contribution from electron excitations to the antibonding σ* d_*x*^2^−*y*^2^_ orbital. The negative contributions from the d_*xy*_ and d_*z*^2^_ molecular orbitals can be interpreted as a 2p → 3d transition accompanied by simultaneous 3d → 3d transitions *i.e.* metal-to-metal charge transfer.

### Partial ion yield (PIY) spectra of CoPPIX^+^

**Fig. 6:**
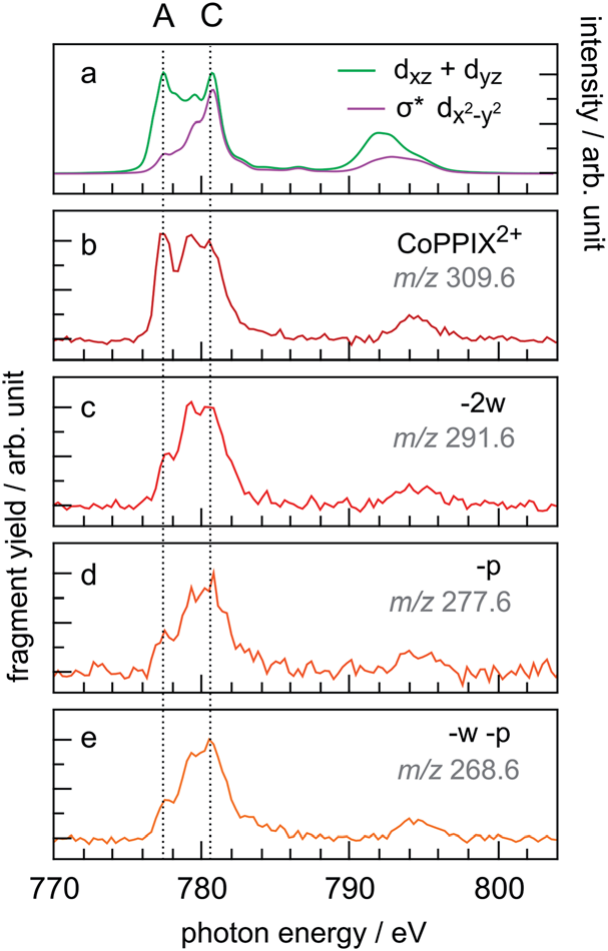
(a) Calculated two main orbital contributions to the cobalt L-edge XAS of CoPPIX^+^ (see Fig. 5). (b–e) Measured PIY spectra of product ions produced after single Auger decay following inner-shell excitation of cobalt 2p electrons of CoPPIX^+^ in the photon energy range 770–804 eV. For better comparison the PIY spectra are normalised to the maximum of peak C.

The PIY spectra for the radical dication CoPPIX^2+^ and for several fragments [CoPPIX–neutral losses]^2+^ formed upon single ionisation are presented in Fig. 6. Only PIYs for fragments well separated from neighbouring peaks (at *m*/*z* > 247) and which can be unambiguously assigned are presented. Other PIYs can be found in Fig. ESI-4.† For comparison, the two calculated main orbital contributions from Fig. 5 are displayed in Fig. 6a.

The PIY spectra in Fig. 6 show a distinct dependence on the resonances in the photon energy range 770–804 eV. The main difference in the PIYs is reflected in an increase of the relative yield at peak A for the intact radical dication CoPPIX^2+^ (Fig. 6b) compared to the fragments formed by neutral losses (Fig. 6c–e). This indicates that resonant excitations at peak A lead to more effective non-dissociative ionisation than fragmentation upon single ionisation. For peak B effects are not as pronounced and we therefore focus on peak A and C in the discussion.

**Fig. 7:**
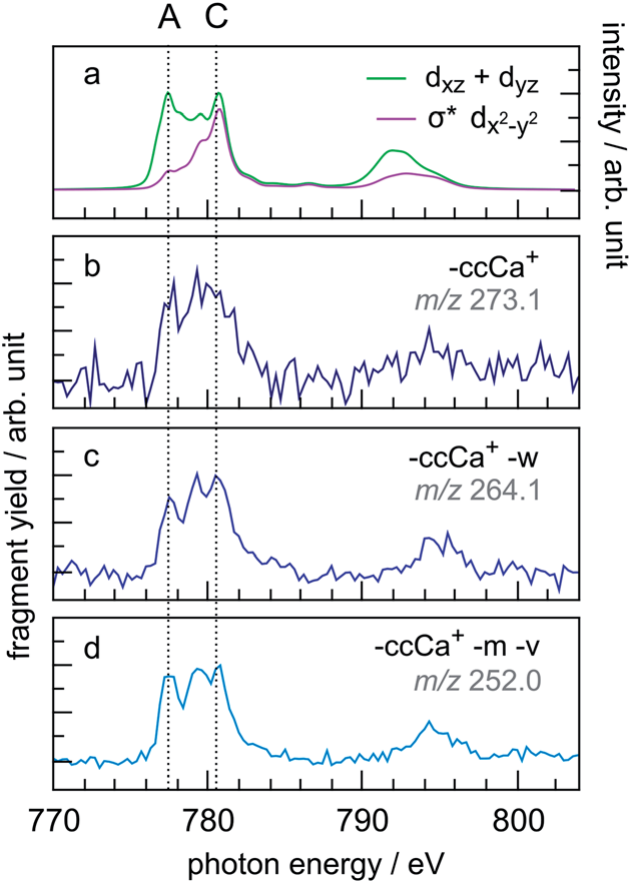
(a) Calculated two main orbital contributions to the cobalt L-edge XAS of CoPPIX^+^ (see Fig. 5). (b–d) Measured PIY spectra of product ions produced upon multiple ionisation following inner-shell excitation of cobalt 2p electrons of CoPPIX^+^ in the photon energy range 770–804 eV. For better comparison the PIY spectra are normalised to the maximum of peak C.

A similar trend in the PIYs as for the radical dication (Fig. 6b) is observed for the –ccCa^+^, –ccCa^+^–w and –ccCa^+^–m–v fragments (Fig. 7b–d, respectively), which are formed upon multiple ionisation of CoPPIX^+^. The similar PIYs for the –ccCa^+^, –ccCa^+^–w and –ccCa^+^–m–v fragments can be attributed to the common involvement of the charged fragment loss (here –ccCa^+^). In contrast, PIYs for other fragments involving charged fragment losses (Fig. ESI-4†) exhibit a similar trend as the fragments formed upon single ionisation (Fig. 6c–e). This can be explained by the fact that, as shown in Fig. 3 and in Table ESI-1,† –ccCa^+^ and –cCa˙^+^ containing fragment peaks have several possible contributions from neutral fragment losses formed upon single ionisation. PIYs for fragments involving a charged fragment loss may thus be superimposed by contributions of fragments formed by neutral losses such that no distinct yield increase for peak A is observed for these peaks.

**Scheme 2:**
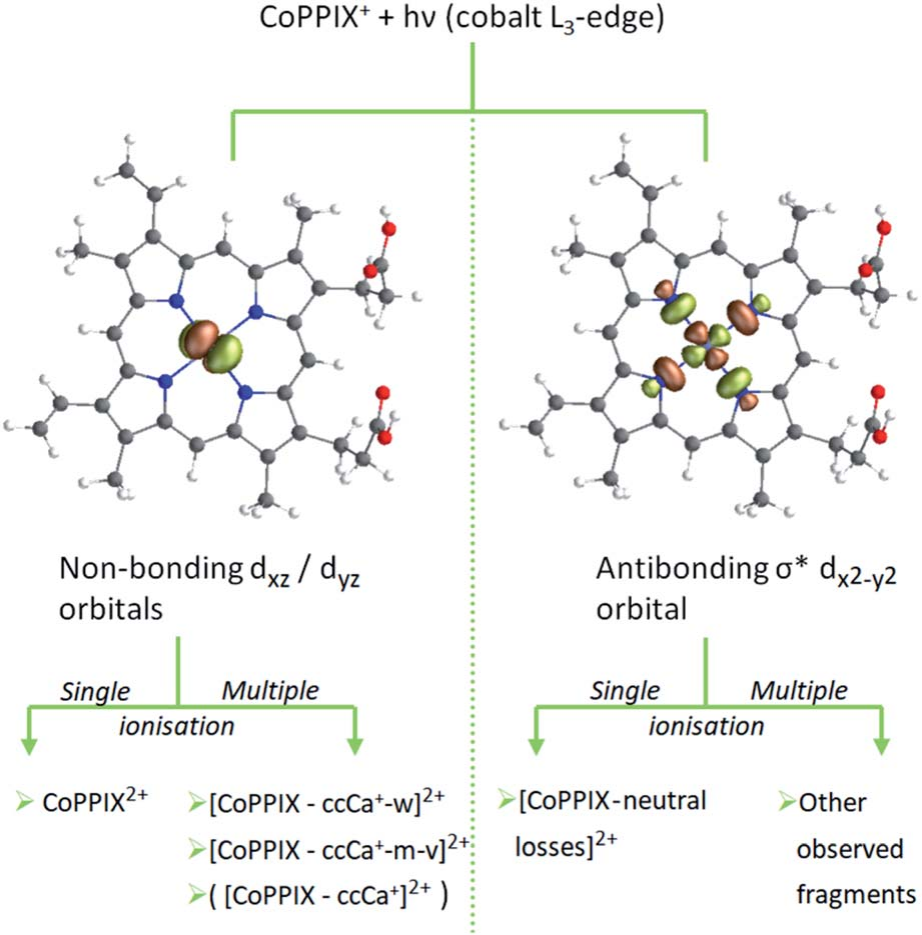
Orbital-specific deexcitation pathways of CoPPIX^+^ after resonant cobalt 2p inner-shell excitation at the L_3_-edge to selected valence molecular orbitals from RAS calculation.

The relative yield increase in peak A for the radical dication and the –ccCa^+^, –ccCa^+^–w and –ccCa^+^–m–v fragments compared to all other observed fragments points to an orbital-specific deexcitation of CoPPIX^+^ after cobalt 2p inner-shell excitation. Based on the orbital-contribution analysis (Fig. 5, 6a and 7a), we assign peak A in the PIY spectra to excitations to predominantly non-bonding d_*xz*_/d_*yz*_ orbitals. Peak C results from excitations to both, non-bonding d_*xz*_/d_*yz*_ orbitals and antibonding σ* d_*x*^2^−*y*^2^_ orbitals. We therefore conclude that cobalt 2p inner-shell excitations to antibonding σ* d_*x*^2^−*y*^2^_ orbitals lead overall to more effective fragmentation compared to excitations to the non-bonding d_*xz*_/d_*yz*_ orbitals, regarding single and multiple ionisation, respectively, which we attribute to the dissociative character of the antibonding orbitals. The results from the PIYs are summarised in Scheme 2, including the observed orbital specificity.

Besides the orbital character, two other effects can contribute to the orbital-specific behaviour. (1) The stabilisation of the core-excited state due to core-hole screening may be different for the two orbital types, thus affecting the deexcitation processes. Indeed, due to the strong metal character of the non-bonding molecular orbitals, we expect that the core-hole screening is stronger for the latter compared to the antibonding orbitals, which can lead to the non-dissociative ionisation upon single ionisation and the comparably weak fragmentation upon multiple ionisation. (2) The internal energies after decay of the cobalt 2p core hole may be different for the different molecular orbitals. A higher internal energy for excitations to the antibonding orbitals compared to the non-bonding orbitals would lead to stronger fragmentation for the former. Besides the superposition of PIYs for fragments from charged and neutral fragment losses with same *m*/*z* (see text above), the difference in internal energies can be the reason for the different PIYs for the –ccCa^+^, –ccCa^+^–w, –ccCa^+^–m–v fragments compared to other fragments involving charged fragment losses. The –ccCa^+^, –ccCa^+^–w, –ccCa^+^–m–v fragments are attributed to excitations to the non-bonding orbitals and accordingly to low-energy deexcitation products of excited [CoPPIX^3+^]*. Other fragments involving –ccCa^+^ and an increased number of side-chain losses, however, would require more internal energy for dissociation thus resembling the contribution of excitations to the antibonding orbitals.

## Conclusions and perspectives

In conclusion, NEXAMS is a suited technique to explore the electronic structure of the metal-active site of isolated CoPPIX^+^ as a “model” metalloporphyrin. In particular, the combination with RAS calculations has proven to be a powerful tool to get insight into the electronic structure of isolated metalloporphyrins and to establish correlations between deexcitation pathways and electronic transitions in such systems. The RAS calculations revealed that the ground state of CoPPIX^+^ is mainly of ^3^A_2g_ triplet character state with a d_*xy*_^2^d_*z*^2^_^2^d_*xz*_^1^d_*yz*_^1^d_*x*^2^−*y*^2^_^0^ configuration with small contributions of other low-lying states. Comparison with the experimental spectrum gave good agreement supporting this theoretical assignment. The further and more accurate determination of the relative energies of the lowest ^3^A_2g_ state to the other low-lying states can be of great interest for both future experimental and theoretical efforts. The current gas-phase experiment on an isolated system proved to be an ideal benchmark for future computations. We found out that deexcitation processes after resonant cobalt 2p inner-shell excitation are orbital specific and that photo products formed by single and multiple ionisation can be distinguished, based on comparison of the partial ion yield (PIY) NEXAMS spectra with the RAS calculations. Here, the high stability of the porphyrin macrocycle towards ionising radiation, and in particular towards core-hole excitation, is remarkable as the CoPPIX^+^ molecule stays either fully intact or relaxes through neutral and charged side-chain losses leaving the macrocycle mostly intact, thus preserving some optical properties of the system.

The following questions now need to be addressed: what are the exact internal energy values and (how) do they affect the deexcitation pathways of metalloporphyrins? How fast is the dissociation from a dissociative state and is it competing with statistical dissociation after ro-vibrational energy redistribution – which would thereby not be quenched in biological media, *i.e.* water? By employing Auger electron spectroscopy and time-resolved NEXAMS on metalloporphyrins it would be possible to access the internal energies and time-scales of decay mechanisms of 2p core excited metalloporphyrins. To date, there is still a lack of dedicated instruments to conduct such experiments on gas-phase electrosprayed ions.

This work is now to be extended to other metalloporphyrin complexes to study the influence of the metal type, its oxidation state and its axial ligands on the electronic structure of the metal centre. Furthermore, electrospray ionisation offers the possibility to exploit the capabilities of NEXAMS by systematically studying metalloporphyrins in bio-relevant systems of increasing complexity in a bottom-up approach. In particular, NEXAMS could be employed to probe the metal-active site in metal-containing proteins, such as hemoglobin and cytochromes. However, this is challenging due to the low contrast in cross sections between resonant photoabsorption in the metal and non-resonant photoabsorption within the protein at the energies of the metal L-edges. This could be overcome by exploiting the metal K-edge, though. Extending the present studies from the “model” metalloporphyrin CoPPIX^+^ to other metalloporphyrin systems will pave the way to a deeper understanding of the properties of metalloporphyrins and metalloporphyrin-containing systems.

## Conflicts of interest

There are no conflicts to declare.

## Supplementary Material

SC-012-D0SC06591A-s001
