# Supplementary material for: The electronic structure and deexcitation pathways of an isolated metalloporphyrin ion resolved by metal L-edge spectroscopy
Source: Chem Sci. 2021 Feb 3;12(11):3966–76. doi: 10.1039/d0sc06591a (PMC8179464; doi:10.1039/d0sc06591a)
Supplement: SC-012-D0SC06591A-s001 [file SC-012-D0SC06591A-s001.pdf]

Electronic Supplementary Material (ESI) for Chemical Science.
This journal is © The Royal Society of Chemistry 2021

The electronic structure and deexcitation pathways of an isolated metalloporphyrin ion resolved by metal L-edge spectroscopy

Electronic Supporting Information

Kaja Schubert,†[a] Meiyuan Guo,†[b#] Kaan Atak,[a] Simon Dörner,[a] Christine Bülow,[c] Bernd von Issendorff,[d] Stephan Klumpp,[a] Tobias Lau,[c,d] Piter S. Miedema,[a] Thomas Schlathölter,[e] Simone Techert,[a,f] Martin Timm,[c] Xin Wang,[e] Vicente Zamudio-Bayer,[c] Lucas Schwob,[a] and Sadia Bari[a]

[a] Deutsches Elektronen-Synchrotron DESY, 22607 Hamburg, Germany. Email: kaja.schubert@desy.de, sadia.bari@desy.de

[b] Division of Chemical Physics, Chemical Center, Lund University, SE-221 00 Lund, Sweden

[c] Abteilung für Hochempfindliche Röntgenspektroskopie, Helmholtz-Zentrum Berlin für Materialien und Energie, 12489 Berlin, Germany

[d] Physikalisches Institut, Albert-Ludwigs-Universität Freiburg, 79104 Freiburg, Germany

[e] Zernike Institute for Advanced Materials, University of Groningen, 9747 AG Groningen, The Netherlands

[f] Institut für Röntgenphysik, Georg-August-Universität Göttingen, 37077 Göttingen, Germany

[#] Current address: SSRL, SLAC National Accelerator Laboratory, Menlo Park, California 94025, USA

† These authors contributed equally to this work.

Experimental Part

The mass calibration was obtained from experiments carried out at the VUV U125-2_NIM beamline (6 - 40 eV) of the BESSY II synchrotron (HZB, Berlin) using the home-built tandem mass spectrometer of the Groningen University group.[1] Similar to the Nanocluster trap end station, cobalt(III) protoporphyrin cations ($CoPPIX^+$) were produced with an electrospray ionisation source, mass-to charge selected and thermalised with helium buffer gas in a Paul trap at room temperature. The ions were accumulated and subsequently irradiated in the ion trap with the VUV photons. Cationic fragments were analysed using a time-of-flight spectrometer in reflectron mode. In contrast to the Nanocluster trap end station, the tandem-mass-spectrometer setup allowed $CoPPIX^+$ precursor ions (*m/z* 619.6) and cationic products at $m/z \gtrapprox 80$ to be detected at the same time and with a spectrometer resolution of $m/\Delta m = 2000$. With this spectrometer configuration, isotopic patterns of singly and doubly charged precursors ions and fragments were resolved.

The mass calibration was based on the well-known photofragmentation mass spectrum of leucine enkephalin, including the parent ion peak (*m/z* 556.3), the b_4 fragment (*m/z* 425.2) and the phenylalanine and tyrosine immonium ions (*m/z* 120.1 and 136.1, respectively).[1,2] VUV photoabsorption of $CoPPIX^+$ (data not shown) leads to photoionisation and to formation of a number of photofragments similar to those observed upon cobalt L-edge photoabsorption of $CoPPIX^+$. Therefore, based on this calibration, the oxidation state and photofragments of $CoPPIX^+$ were assigned (see main text) and a mass calibration for the Nanocluster trap end station was obtained.

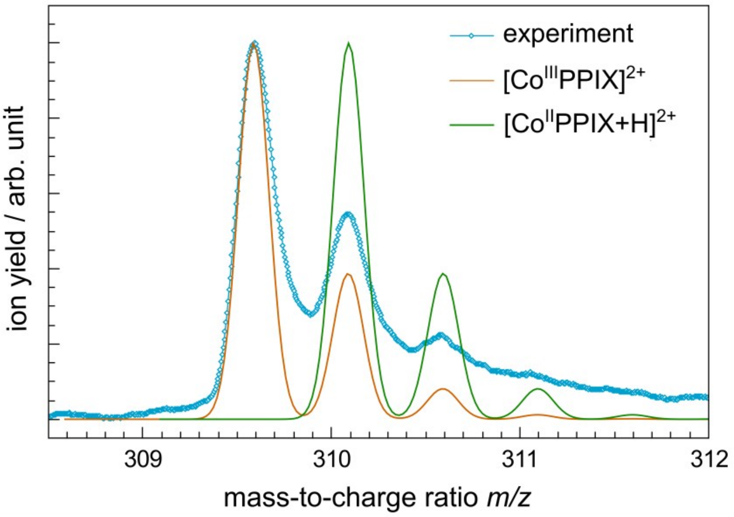

Figure ESI-1 Theoretical isotopic patterns of $Co^{III}PPIX^{2+}$ (orange line) and $[Co^{II}PPIX + H]^{2+}$ (green line) in comparison with our measured mass spectrum after resonant excitation at 780.5 eV at the cobalt L_3-edge (blue diamonds). The isotopic patterns are broadened with our experimental resolution of $m/\Delta m = 1600$ with a Gaussian function to model the peak shapes in our mass spectrum.

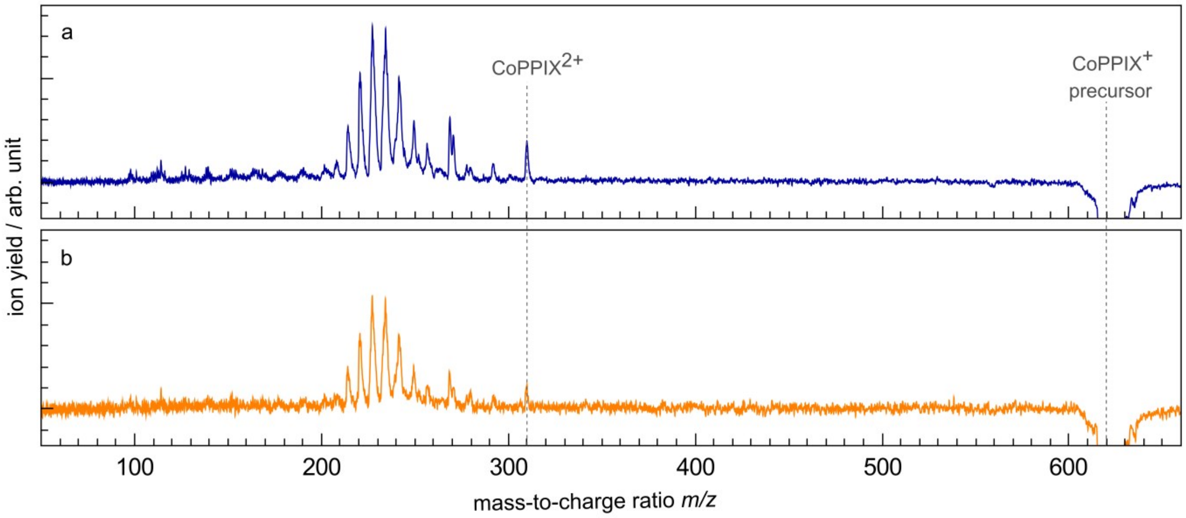

Figure ESI-2 Mass spectrum of $CoPPIX^{+}$ after a. resonant photoabsorption at the cobalt L_3-edge (778 eV) and b. non-resonant photoabsorption below the cobalt L_3-edge (770 eV), measured with the tandem mass spectrometer setup at room temperature at the P04 beamline. The precursor ion peaks appear negative in the spectrum due to the data treatment.

Similar measurements at the L_3-edge as with the Nanocluster trap end station were performed at the P04 beamline[3] at the PETRA III synchrotron (DESY, Hamburg) with the home-built tandem mass spectrometer of the Groningen University group (see above) albeit with the time-of-flight mass spectrometer in linear mode ($m/\Delta m$ = 260). The exit slits and grating of the monochromator of the beamline were set to an energy bandwidth of 4.5 eV at 770 eV.

Figure ESI-2 shows the mass spectrum of $CoPPIX^{+}$ ions obtained after resonant photoexcitation at the cobalt L_3-edge. In contrast to the Nanocluster trap end station (see main text) and as explained earlier, the tandem mass spectrometer enables the trapping and detection of ions over a broad mass range covering the region where singly, doubly and triply charged fragment peaks are expected (m/z < 610, m/z < 310 and m/z < 206, respectively). Due to the data treatment the peak corresponding to the precursor ions at m/z 620 appears negative reflecting the depletion of precursor ions upon irradiation. The present data show no evidence for the production of singly charged fragments and we thus assume that singly charged fragments are not produced during the experiments performed at the Nanocluster trap end station.

The fragmentation pattern in Figure ESI-2 is similar as in Figure 2, although the mass resolution in Figure 2 allows a better differentiation of the fragment peaks (see Experimental section). The $CoPPIX^{2+}$ signal is about twice as high than the signal of fragments with 200 < m/z < 250 in Figure 2 compared to Figure ESI-2. One reason for the difference can be the trap and the spectrometer settings for the Nanocluster trap end station, which were set for a higher sensitivity to the high m/z range. Another reason can be the difference in temperature in both experiments. Data in Figure ESI-2 were collected at room temperature, whereas data in Figure 2 were collected at 18K *i.e.* with lower internal energy. Additionally, for the Nanocluster trap the helium buffer gas is constantly present during the irradiation such that irradiated precursor ions may cool down excess energy by collision with the helium which could prevent some fragmentation. This was not the case for the experiments presented in Figure ESI-2 where the helium injection in the trap is pulsed synchronously to the ion injection and pumped down before irradiation.

m/z	Losses of $CoPPIX^{2+/3+}$				
300.6	-w				
291.6	-2w				
280.1	-cCa$^{\bullet}$				
279.6	-m-w-v	-4m	-cCa$^{\bullet+}$	-m-Ca	
278.1	-2w-v	-3m-w			
277.6	-p				
273.1	-ccCa^{+}	-ccCa$^{\bullet\&}$			
270.6	-m-2w-v	-4m-w	-cCa$^{\bullet+}$-w	-m-Ca-w	
268.6	-w-p				
264.1	-ccCa^{+} -w	-ccCa$^{\bullet}$ -w$^{\&}$	-v-p$^{\&}$		
258.5	-2m-w-2v	-m-cCa$^{\bullet+}$-v	-2m-Ca-v		
257.1	-m-2w-2v	-4m-w-v	-cCa$^{\bullet+}$-w-v	-3m-cCa$^{\bullet+}$	-m-Ca-w-v
256.5	-m-ccCa$^{+/\bullet}$-w	-m-v-p			
252.1	-m-ccCa^{+}-v	-m-ccCa$^{\bullet}$-v$^{\&}$	-m-w-2p$^{\&}$		
250.1	-4m-cCa$^{\bullet}$	-m-cCa$^{\bullet}$-w-v	-cCa$^{\bullet+}$-cCa$^{\bullet}$	-m-Ca-cCa$^{\bullet}$	
249.5	-2m-2w-2v	-4m-cCa$^{\bullet+}$	-m-cCa$^{\bullet+}$-w-v	-2m-Ca-w-v	
249.1	-2m-ccCa$^{+/\bullet}$-w	-2m-v-p			
248.5	-3m-cCa$^{\bullet}$-w				
244.5	-2m-2w-p	-2m-ccCa$^{+/\bullet}$-v			
243.0	-cCa$^{\bullet+}$-ccCa$^{\bullet}$	-m-2cCa$^{\bullet}$	-m-ccCa$^{+/\bullet}$-w-v	-4m-ccCa$^{+/\bullet}$	-m-Ca-ccCa$^{+/\bullet}$
242.5	-2m-cCa$^{\bullet}$-w-v	-m-cCa$^{\bullet}$-cCa$^{\bullet+}$	-2m-cCa$^{\bullet}$-Ca		
242.0	-2m-cCa$^{\bullet+}$-w-v	-3m-2w-2v	-3m-Ca-w-v		
241.5	-3m-ccCa$^{+/\bullet}$-w	-3m-v-p			
241.0	-ccCa$^{+/\bullet}$-p	-4m-cCa$^{\bullet}$-w			
240.5	-m-cCa$^{\bullet}$-p	-4m-cCa$^{\bullet+}$-w			
235.5	-m-cCa$^{\bullet+}$-ccCa$^{\bullet}$	-2m-2cCa$^{\bullet}$	-2m-ccCa$^{+/\bullet}$-w-v	-2m-Ca-ccCa$^{+/\bullet}$	
235.0	-3m-cCa$^{\bullet}$-w-v				
234.5	-3m-cCa$^{\bullet+}$-w-v	-cCa$^{\bullet}$-v-p	-4m-2w-2v	-4m-Ca-w-v	
234.0	-4m-ccCa$^{+/\bullet}$-w	-cCa$^{\bullet+}$-v-p	-m-Ca-v-p		
228.5	-2m-cCa$^{\bullet+}$-w-2v	-2m-cCa$^{\bullet}$-ccCa$^{+/\bullet}$	-3m-Ca-w-2v		
228.0	-3m-2cCa$^{\bullet}$	-3m-ccCa$^{+/\bullet}$-w-v			
227.5	-3m-cCa$^{\bullet+}$-cCa$^{\bullet}$	-2w-2p	-ccCa$^{+/\bullet}$-v-p	-4m-cCa$^{\bullet}$-w-v	-4m-Ca-cCa$^{\bullet}$
227.0	-m-cCa$^{\bullet}$-v-p	-4m-cCa$^{\bullet+}$-w-v			
221.5	-2m-ccCa$^{+/\bullet}$-cCa$^{\bullet}$	-2m-cCa$^{\bullet+}$-cCa$^{\bullet}$-v	-3m-cCa$^{\bullet}$-w-2v	-3m-Ca-cCa$^{\bullet}$-v	
221.0	-3m-ccCa$^{+/\bullet}$-cCa$^{\bullet}$	-3m-cCa$^{+\bullet}$-w-2v	-4m-Ca-w-2v		
220.5	-3m-ccCa$^{\bullet}$-cCa$^{\bullet+}$	-4m-2cCa$^{\bullet}$	-4m-ccCa$^{+/\bullet}$-w-v	-4m-ccCa$^{\bullet/+}$-Ca	
215.0	-2m-cCa$^{\bullet}$-ccCa$^{+/\bullet}$-v				
214.5	-2m-cCa$^{+\bullet}$-ccCa$^{\bullet}$-v	-3m-ccCa$^{+/\bullet}$-w-2v	-3m-2cCa$^{\bullet}$-v	-3m-Ca-ccCa$^{\bullet/+}$-v	
214.0	-3m-2ccCa$^{+/\bullet}$	-3m-cCa$^{\bullet}$-cCa$^{+\bullet}$-v	-4m-cCa$^{\bullet}$-w-2v	-4m-cCa$^{\bullet}$-Ca-v	

Table ESI-1 Possible assignments of peaks observed in the mass spectrum of $CoPPIX^{+}$ upon resonant absorption at the cobalt L_3-edge in the *m/z*-range of doubly charged fragments. Note that isotopes can additionally contribute to the listed peaks. Contributions of fragments marked with "&" are negligible (see main text and Figure 4).

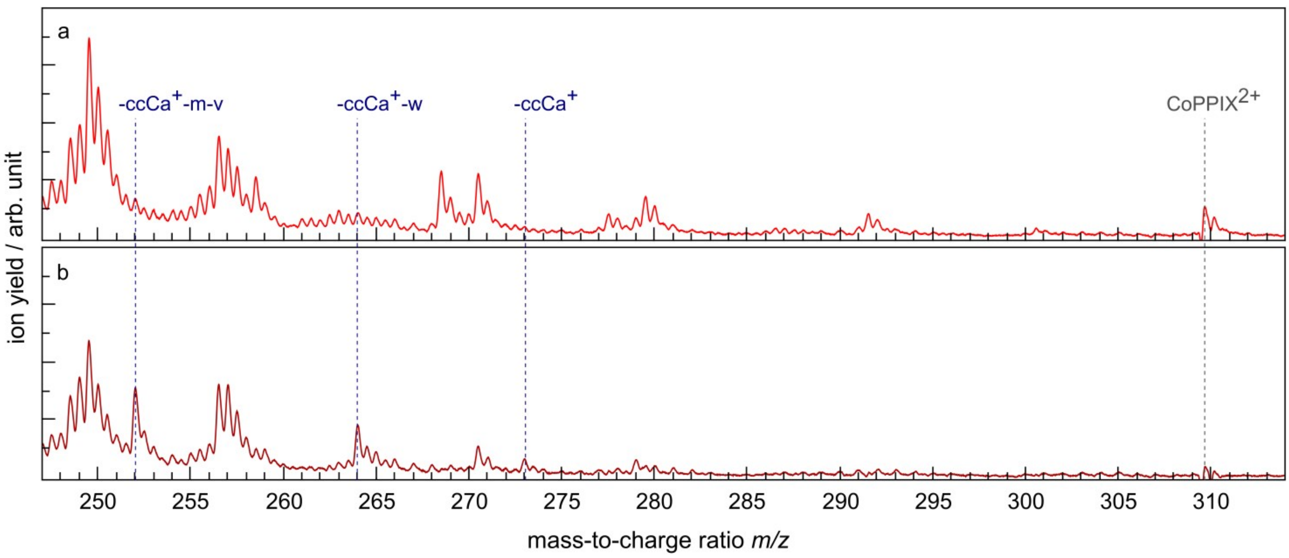

Figure ESI-3 Mass spectrum of CoPPIX^{+} after irradiation in the energy region of the carbon K-edge for a. resonant excitation at 288.6 eV and for b. photoionisation at 298.1 eV measured at the Nanocluster trap end station.

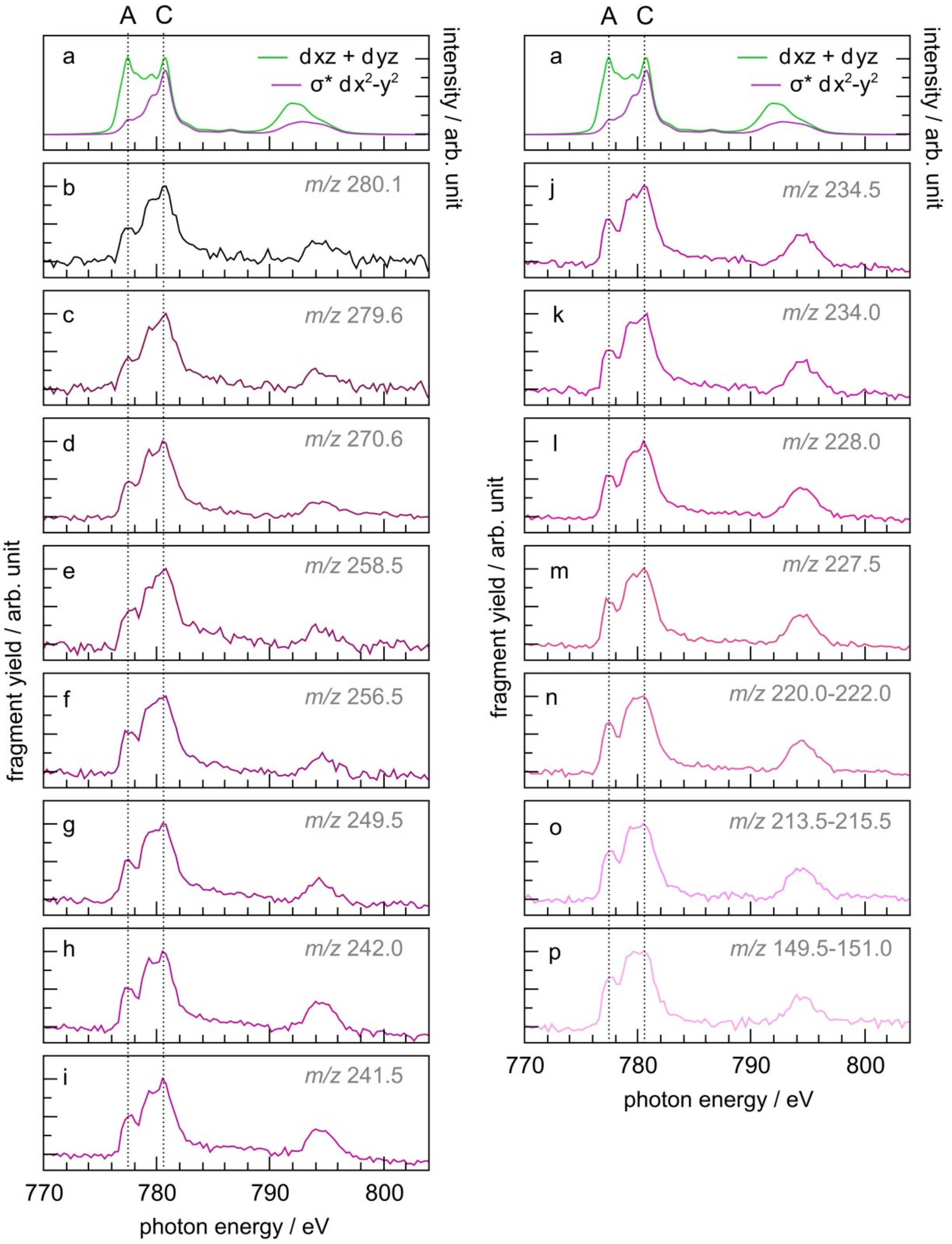

Figure ESI-4 a. Calculated two main orbital contributions to the cobalt L-edge XAS of $CoPPIX^{+}$ (see Figure 5). b-p. Measured PIY spectra for several product ions formed after inner-shell excitation of cobalt 2p electrons of $CoPPIX^{+}$ in the photon energy range 770-804 eV. The PIYs of these photofragments are partly affected by contributions resulting from overlapping peaks in the mass spectrum, multiple possible fragments of same *m/z* (see Table ESI-1) and isotopic contributions from neighboring fragment peaks. For better comparison the PIY spectra are normalised to the maximum of peak C.

Computational Part

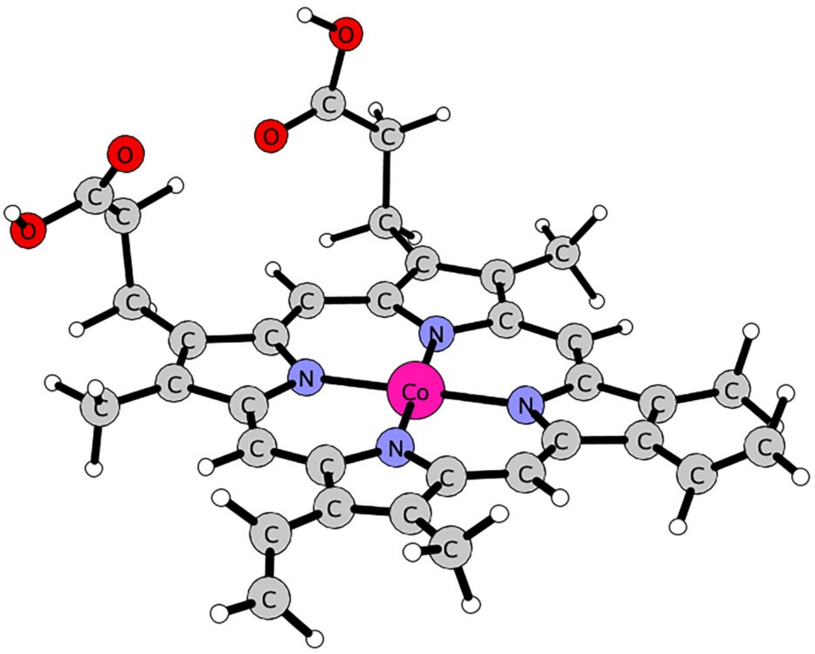

Figure ESI-5 Calculated structure of $CoPPIX^{+}$ after geometry optimisation using density functional theory (DFT) with the B3LYP functional and the 6-311g* basis set using Gaussian 09.[4]

atom	x	y	z
C	-3.94318	2.88474	0.34251
C	-3.42816	1.52835	0.22646
C	-4.2159	0.39853	0.37385
C	-3.75943	-0.89402	0.22045
C	-4.62784	-2.07636	0.34012
C	-5.34674	3.23834	0.71359
C	-6.06282	-2.0133	0.59813
C	-6.75834	-2.87604	1.34657
N	-2.46422	-1.25261	-0.04714
H	-5.26092	0.54114	0.61004
H	-6.59929	-1.18645	0.13744
H	-7.82904	-2.76579	1.47468
H	-6.2894	-3.6969	1.87734
H	-5.39871	4.25716	1.09926
H	-6.02291	3.1797	-0.14623
H	-5.74156	2.57145	1.48358
C	-2.888	3.71393	0.08903
N	-2.0854	1.51943	-0.06706
C	-3.82691	-3.15656	0.12559
C	-2.48647	-2.62541	-0.10303
C	-2.82812	5.17197	0.08348
C	-1.73348	2.84297	-0.15349
C	-4.19211	-4.60439	0.07124
C	-1.38265	-3.4285	-0.33343
C	-3.79291	5.98944	-0.35149
H	-1.91226	5.61786	0.46512
C	-0.4672	3.31023	-0.43677
H	-3.68375	-5.11902	-0.74789
H	-5.26554	-4.7263	-0.07714
C	-0.09894	-2.95738	-0.52194
H	-1.53234	-4.49938	-0.34592
H	-3.67262	7.06581	-0.3082
H	-4.71454	5.62289	-0.7891
H	-0.32516	4.37934	-0.51052
C	0.6419	2.49951	-0.61281
C	1.05127	-3.82937	-0.73366
N	0.27352	-1.63509	-0.52722
C	1.98934	3.03248	-0.83797

atom	x	y	z
N	0.64091	1.13306	-0.56656
C	2.13449	-3.01659	-0.85614
C	0.98395	-5.32134	-0.80253
C	1.63299	-1.64774	-0.72704
C	2.81448	1.95925	-0.90633
C	2.30339	4.48835	-0.96441
C	1.95872	0.77682	-0.74822
C	3.55623	-3.44277	-1.10606
H	1.95136	-5.75514	-1.0557
H	0.26743	-5.65786	-1.55763
H	0.67324	-5.75521	0.15341
C	2.43037	-0.51816	-0.81735
C	4.30262	1.9343	-1.10375
H	3.37066	4.65693	-1.09424
H	1.78281	4.93541	-1.8174
H	1.99688	5.04198	-0.07133
H	4.12552	-2.64168	-1.57947
H	3.54697	-4.2661	-1.825
C	4.31183	-3.91506	0.15027
H	3.49369	-0.66588	-0.92926
H	4.62342	2.87842	-1.54578
H	4.5701	1.15742	-1.82638
C	5.11583	1.68034	0.18858
C	4.80788	-2.78159	1.01999
H	3.71344	-4.59661	0.75783
H	5.20107	-4.48159	-0.15014
H	4.8831	0.71371	0.62991
H	6.17815	1.67194	-0.07677
C	4.91676	2.72907	1.2577
O	4.9959	-1.64787	0.64463
O	5.05916	-3.18959	2.27293
O	5.20315	3.97675	0.80154
O	4.56254	2.52241	2.38834
H	-3.92841	-5.12706	0.99707
H	5.41808	-2.43861	2.77208
H	5.09199	4.58774	1.54648
Co	-0.90772	-0.05892	-0.3015

Table ESI-2 Coordinates of $CoPPIX^{+}$ after geometry optimisation using density functional theory (DFT) with the B3LYP functional and the 6-311g* basis set using Gaussian 09.[4]

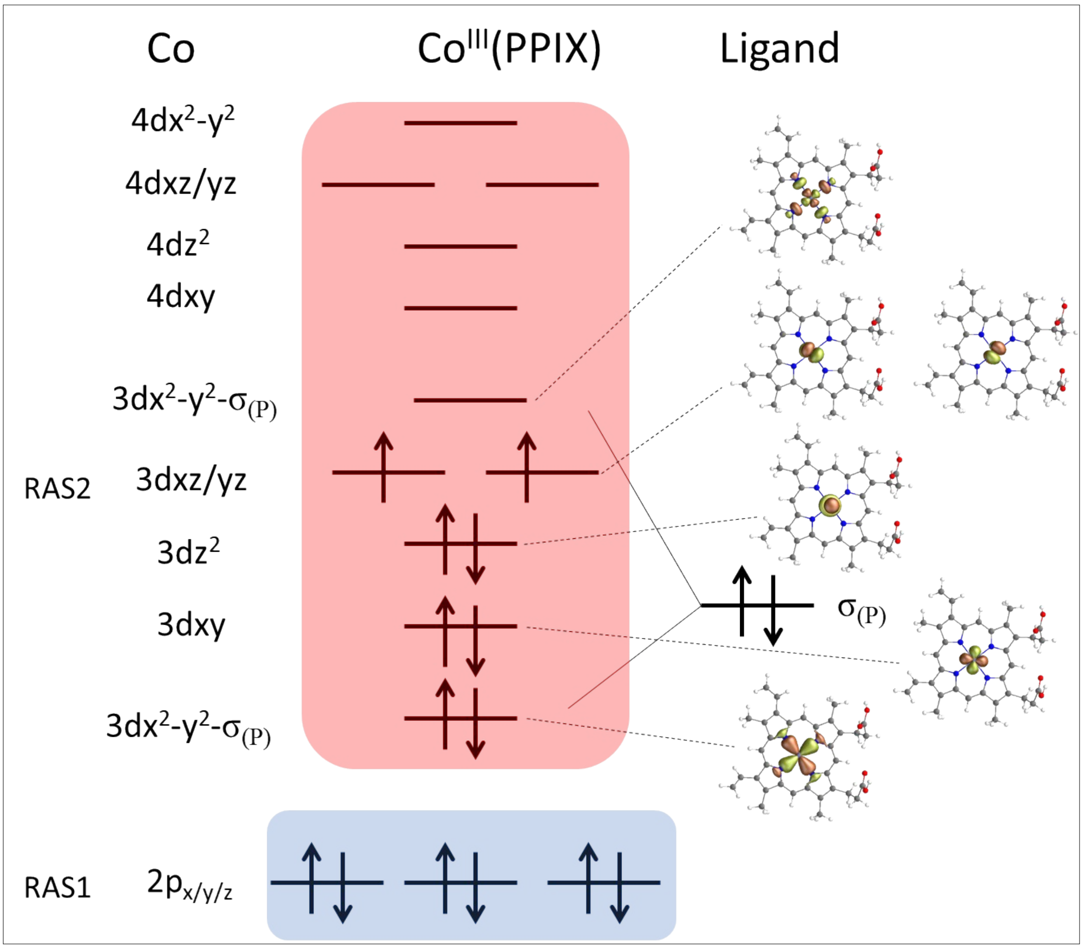

Figure ESI-6 Active space and selected active orbitals for the restricted active space calculations of cobalt L-edge XAS.

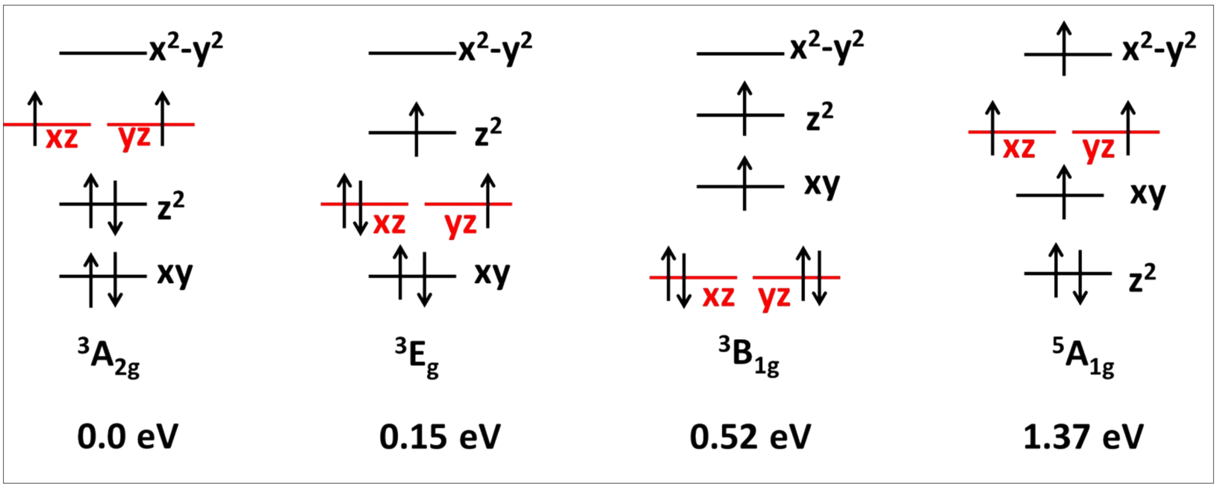

Figure ESI-7 Schematic representations of selected low-lying states in $CoPPIX^+$ which were used for X-ray spectra simulations. The relative energy is shown by taking lowest energy state $^3A_{2g}$ as reference.

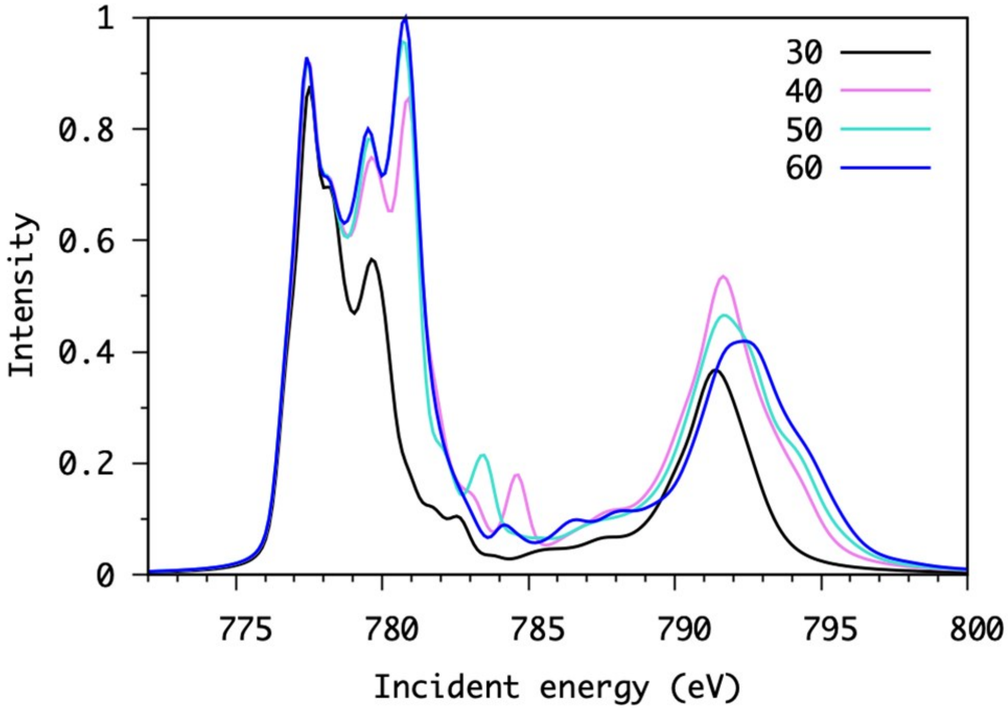

Figure ESI-8 The dependence on the number of core excited states for each spin state for the $^3A_{2g}$ ground state.

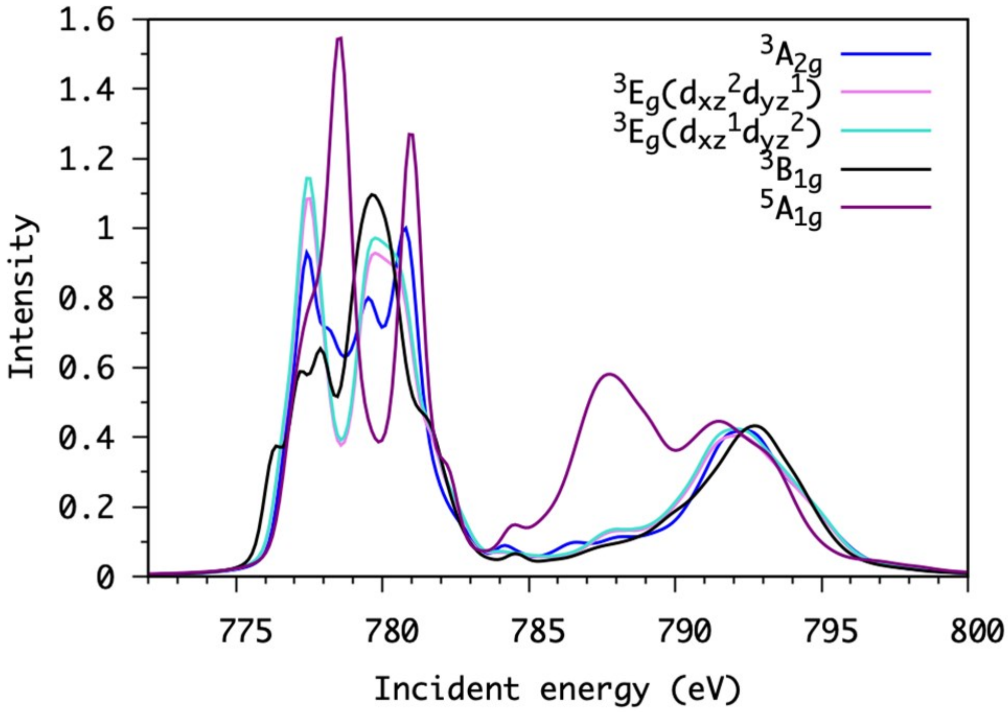

Figure ESI-9 The XAS calculation with different initial states having different electronic configuration as presented in the Figure ESI-7.

	$^3A_{2g}$	$^3E_g(dxz^2dyz^1)$	$^3E_g(dxz^1dyz^2)$	$^3B_{1g}$	$^5A_{1g}$
Similarity score	0.9366	0.9205	0.9194	0.9334	0.8596

Table ESI-3 Similarity scores for the cobalt L-edge XAS spectra calculated with different initial state configurations and compared to the experimental TIY spectrum, analysed through a weighted cross-correlation function.[5]

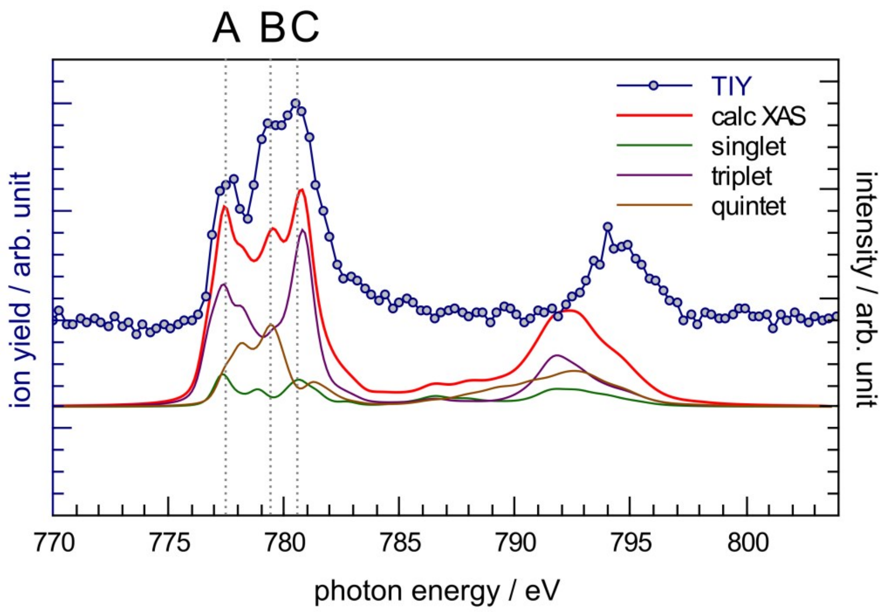

Figure ESI-10 Comparison between the experimental TIY of $CoPPIX^+$ and calculated XAS spectra at the cobalt L-edge including the core-excited states contribution analysis.

References

1 S. Bari, O. González-Magaña, G. Reitsma, J. Werner, S. Schippers, R. Hoekstra and T. Schlathölter, *J. Chem. Phys.*, 2011, **134**, 024314.

2 M. L. J. Ranković, F. Canon, L. Nahon, A. Giuliani and A. R. Milosavljević, *J. Chem. Phys.*, 2015, **143**, 244311.

3 J. Viefhaus, F. Scholz, S. Deinert, L. Glaser, M. Ilchen, J. Seltmann, P. Walter and F. Siewert, *Nucl. Instruments Methods Phys. Res. Sect. A Accel. Spectrometers, Detect. Assoc. Equip.*, 2013, **710**, 151–154.

4 M. J. Frisch, G. W. Trucks, H. B. Schlegel, G. E. Scuseria, M. A. Robb, J. R. Cheeseman, G. Scalmani, V. Barone, B. Mennucci, G. A. Petersson, H. Nakatsuji, M. Caricato, X. Li, H. P. Hratchian, A. F. Izmaylov, J. Bloino, G. Zheng, J. L. Sonnenberg, M. Hada, M. Ehara, K. Toyota, R. Fukuda, J. Hasegawa, M. Ishida, T. Nakajima, Y. Honda, O. Kitao, H. Nakai, T. Vreven, J. A. Montgomery, J. E. Peralta, F. Ogliaro, M. Bearpark, J. J. Heyd, E. Brothers, K. N. Kudin, V. N. Staroverov, R. Kobayashi, J. Normand, K. Raghavachari, A. Rendell, J. C. Burant, S. S. Iyengar, J. Tomasi, M. Cossi, N. Rega, J. M. Millam, M. Klene, J. E. Knox, J. B. Cross, V. Bakken, C. Adamo, J. Jaramillo, R. Gomperts, R. E. Stratmann, O. Yazyev, A. J. Austin, R. Cammi, C. Pomelli, J. W. Ochterski, R. L. Martin, K. Morokuma, V. G. Zakrzewski, G. A. Voth, P. Salvador, J. J. Dannenberg, S. Dapprich, A. D. Daniels, Ö. Farkas, J. B. Foresman, J. V Ortiz, J. Cioslowski and D. J. Fox, Gaussian 09 (Revision A.02), Gaussian Inc., Wallingford CT, 2009.

5 E. Källman, M. G. Delcey, M. Guo, R. Lindh and M. Lundberg, *Chem. Phys.*, 2020, **535**, 110786.
